# Laparoscopic donor right hepatectomy in a donor with type III portal vein anomaly: A case report

**DOI:** 10.1097/MD.0000000000016736

**Published:** 2019-08-09

**Authors:** Jiu-Lin Song, Hong Wu, Jia-Yin Yang

**Affiliations:** Liver Transplantation Center, Department of Liver Surgery, West China Hospital of Sichuan University, Chengdu, Sichuan Province, China.

**Keywords:** anomalous portal vein branching, laparoscopic donor right hepatectomy, living donor liver transplantation, reconstruction of portal vein

## Abstract

**Rationale:**

Laparoscopic right donor hepatectomy has been reported sporadically in several experienced centers for selected donors. This report introduced a case of a donor with an independent right posterior segmental portal branching from the main portal vein.

**Patient concerns:**

A 47-year-old woman volunteered to donate her right liver to her 48-year-old husband.

**Diagnoses:**

The recipient has been diagnosed as hepatocellular carcinoma meeting the Milan criteria and hepatitis B virus related cirrhosis.

**Interventions:**

The parenchymal transection was performed by ultrasonic aspirator and Hem-o-Lok clips. The right hepatic artery, right hepatic duct, and the anterior and posterior branches of right portal vein were meticulously dissected, clamped, and transected. The right hepatic vein was transected by vascular stapler. A Y-graft of the recipient's own portal confluence was reconstructed with the donor's separate right anterior and posterior portal veins.

**Outcomes:**

The donor's operation time was 420 minutes and the warm ischemia time was about 9 minutes. Blood loss was less than 600 ml without transfusion. The donor was discharged at the 10th postoperative day without any complications.

**Lessons:**

Laparoscopic right hepatectomy for donors with anomalous portal vein branching and subsequent inflow reconstruction for adult living donor liver transplantation is safe and feasible in highly experienced center.

## 1 Introduction

Laparoscopic donor hepatectomy for living donor liver transplantation (LDLT) has been widely accepted throughout the world in experienced centers since the first report in 2002.^[1]^ Though the laparoscopic approach has been widely applied for procurement of the left lateral liver graft,^[2]^ laparoscopic donor right hepatectomy (LDRH) remains challenging.^[3]^ Concerning the donor's safety, the LDRH was principally recommended for selected donors with normal anatomy of vessels.^[4]^ For expanding the donor pool for laparoscopic donor hepatectomy, we reported a case of LDRH for a donor with a type III portal vein anatomy (an independent right posterior segmental portal branching from the main portal vein) which was reconstructed with a Y-graft of the recipient's own portal confluence.

## 2 Case presentation

A 47-year-old woman (weight: 62 kg; height: 159 cm; blood type: O) volunteered to donate her right liver to her 48-year-old husband (weight: 60 kg; height: 168 cm). The recipient was diagnosed as hepatocellular carcinoma meeting the Milan criteria and hepatitis B virus related cirrhosis, with a Child-Pugh score of 10, and a model for end-stage liver disease score of 13.

**Figure 1:**
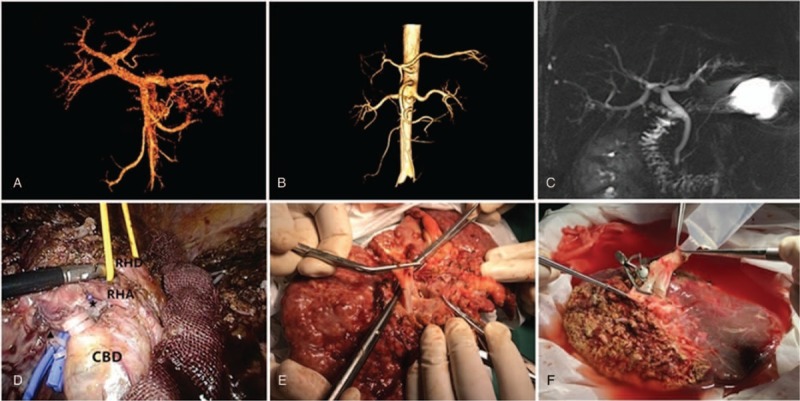
**(A)** Anatomy of donor's portal vein by CT scan. **(B)** Anatomy of donor's hepatic artery by CT scan. **(C)** Anatomy of donor's biliary tree by MRCP. **(D)** Dissection of portal veins (★: right anterior portal vein; ▴: right posterior portal vein), hepatic artery and bile duct. RHA: right hepatic artery; RHD: right hepatic duct; CBD: common bile duct. **(E)** The Y-graft of the recipient's own portal confluence. **(F)** Right liver graft with reconstruction of separate right anterior and posterior portal veins using a Y-graft of the recipient's own portal confluence.

The donor was comprehensively evaluated before operation. The preoperative computed tomography (CT) showed that the donor had a type III portal vein anatomy with an independent right posterior segmental portal branching from the main portal vein (Fig. 1A). However, the CT and the magnetic resonance cholangiopancreatography (MRCP) showed that the right liver had a single right hepatic artery (RHA, Fig. 1B) and a single right hepatic duct (RHD, Fig. 1C). And the CT volumetry showed that the donor's right liver volume, without the middle hepatic vein (HMV), was 689 cc, the graft to recipient weight ratio (GRWR) was 1.15% and the remnant liver volume rate was 36.6%.

Both the donor and recipient were informed about the risks of laparoscopic approaches in LDLT, and the written informed consents of LDRH were obtained. This donation was registered in China Liver Transplantation Register (http://www.cltr.org/). All the procedures performed in this case were in accordance with the ethical principles of the Helsinki Declaration and approved by the Ethics Committee of the West China Hospital of Sichuan University.

The donor was put in a 30° reverse Trendelenburg position with arms and legs abducted. The pneumoperitoneum was established at 13 mm Hg and 5 laparoscopic trocars were inserted as usual.^[5]^ The donor has received cholecystectomy 1 year ago. After total mobilization of the right liver, the right hepatic vein (RHV) was dissected and encircled with a silicone tube for hanging maneuver. Then, the right hepatic pedicle was dissected, and the RHA and right portal veins (RPV) were encircled (Fig. 1D).

The liver parenchyma was transected with a laparoscopic Harmonic^TM^ scalpel (© Ethicon, Somerville, NJ, USA) and an ultrasonic aspirator (CUSA Excel+, Integra, New Jersey, USA). The liver capsule transection line was along with the ischemic demarcation line by transiently clamping the RHA and RPVs. The laparoscopic ultrasound was used to identify MHV, which was reserved for the donor. The intrahepatic vessels were divided and sealed with a LigaSure^TM^ dolphin tip 37 cm laparoscopic instrument (LS1500, © Medtronic, Minneapolis, USA) between Hem-o-Lok clips (Weck, Telefex Medical, North Carolina, USA).

After the parenchymal transection was completed, the RHD was clamped and divided at the appreciate point after rereading the MRCP images and performing intraoperative roentgenographic cholangiography. After a 10-cm caesarean incision was prepared and the heparin was injected, the RHA and RPVs were clamped and transected. The RHV was transected using a vascular stapler. The right liver was extracted from the suprapubic incision.

The graft was perfused with 2000 ml of HTK solution immediately. The graft had separate openings of right anterior and right posterior portal veins. A Y-graft interposition technique using the recipient's own portal confluence (Fig. 1E) was applied for reconstruction of the graft's portal veins at the back table (Fig. 1F). Then, the opening of the Y-graft was anastomosed with the recipient's main portal vein.

The donor's operation time was 420 minutes. The warm ischemia time was about 9 minutes. The blood loss was less than 600 ml without transfusion and anotherintraoperative complications. The graft weighted 500 g with a GRWR of 0.83%. The donor was discharged at the 10th postoperative day uneventfully without postoperative complications. The recipient was discharged at the 8th postoperative day with normal graft function.

## 3 Discussion

LDLT has become an alternative to deceased donor liver transplantation for patients with end-stage liver disease.^[6]^ However, the donors substantially suffered from medical, social, and psychological burden of heavy laparotomy trauma after donor hepatectomy.^[7]^ Application of laparoscopic procedures for donor hepatectomy with caesarean incision could alleviate the postoperative pain, increase the cosmetic satisfaction, and enhance the recovery to normal life.^[8]^ Laparoscopic donor left lateral hepatectomy has been recommended as a new standard practice for pediatric living donor liver transplantation in highly specialized centers.^[2]^ However, the laparoscopic donor major hepatectpomy remains a challenging innovative procedure and requires a high level of surgical skills.^[3]^ Concerning the donor's safety, LDRH was performed in selected donors without anomalies of bike duct or portal vein during a small number of highly experienced centers.^[4,9,10]^

Furthermore, the surgical teams performing laparoscopic donor hepatectomy (especially of LDRH) are recommended for technical expertise of both the LDLT and laparoscopic hepatectomy. From January 2001 to December 2018, 401 LDLTs (including 53 left lateral lobes, 43 left lobes, and 305 right lobes) have been performed in West China hospital. Fortunately, there was no donor death in our center.^[11]^ Laparoscopic hepatectomy was initially performed from 2009 in our center, and the experience of laparoscopic major hepatectomy for cirrhotic patients has been mature since 2015.^[11]^ Meanwhile, several minimally invasive laparoscopic techniques, including laparoscopic assisted technique, hybrid technique, and pure laparoscopy, have been performed for living donor liver grafts harvest in our center.^[5,12,13]^ From October 2015 to December 2018, we have completed 19 laparoscopic living donor hepatectomies, including 6 left lateral lobes, 6 left lobes, and 7 right lobes (including this case).^[5]^

The portal vein variations are infrequent, but of immense clinical significance in right lobe LDLT. The variants of the portal vein were classified as type I (bifurcation), type II (trifurcation), and type III (independent right posterior segmental portal branching from the main portal vein).^[14]^ And the type III portal vein anatomy was discovered in 3.3% to 12.7% of right lobe donors.^[14–16]^ The anomalous portal vein branching (including type II and III portal vein anatomy) may result in 2 portal venous openings in a right lobe graft. The candidates for right liver donation with the anomalous portal vein branching were often disqualified because of the technical difficulty in vascular dissection and reconstruction. One challenging issue for the type III portal vein anatomy is the dissection of anterior portal branch, which is located deeply in the hilum. And inappropriate transection of the anterior and posterior portal branch may injury the reserved left portal vein of donor.^[14]^

With the accumulation of experience, we attempted to perform this LRDH for a donor with type III portal vein anatomy to extend the selection criteria of donors for laparoscopic surgery. For the donors with type III portal vein anatomy, the right anterior and posterior portal veins should be dived separately for the donor's safety.^[14]^ Another challenging issue for the donor with anomalous portal vein branching is the reconstruction of separate portal branches. The separate branches can not be sutured together simply because the 2 graft portal openings are always far apart. And previous study has reported that direct double anastomoses of separate graft's portal branches with recipient's left and right portal veins might cause postoperative portal venous thrombosis.^[14]^ So we performed the double anastomoses with the recipient's portal vein Y-graft on the back table to avoid compromising the donor's reserved portal vein.

In conclusion, we present our initial experience in LRDH for the donor with anomalous portal vein branching. However, it could only be performed by experienced surgical team with expertise of laparoscopic liver surgery, living donor hepatectomy and phleboplasty.

## Acknowledgments

We acknowledged the China Liver Transplant Registry (http://www.cltr.org/) for supporting the study data.

## Author contributions

**Conceptualization:** Jiu-Lin Song.

**Formal analysis:** Jiu-Lin Song.

**Investigation:** Jiu-Lin Song.

**Methodology:** Jiu-Lin Song, Hong Wu, Jia-Yin Yang.

**Resources:** Jia-Yin Yang.

**Supervision:** Hong Wu, Jia-Yin Yang.

**Writing – original draft:** Jiu-Lin Song.

**Writing – review & editing:** Hong Wu, Jia-Yin Yang.

Jiu-Lin Song orcid: 0000-0003-3555-2641.
